# Appetitive information seeking behaviour reveals robust daily rhythmicity for Internet-based food-related keyword searches

**DOI:** 10.1098/rsos.172080

**Published:** 2018-07-25

**Authors:** Nicolas Scrutton Alvarado, Tyler J. Stevenson

**Affiliations:** School of Biological Sciences, University of Aberdeen, Aberdeen, UK

**Keywords:** human, chronotypes, big-data, Google, foraging theory

## Abstract

There has been an exponential growth of information seeking behaviour (ISB) via Internet-based programs over the past decade. The availability of software that record ISB temporal patterns has provided a valuable opportunity to examine biological rhythms in human behaviour. Internet search repositories, such as Google Trends, permit the analyses of large datasets that can be used to track ISB on a domestic and international scale. We examined daily and seasonal Google Trends search patterns for keywords related to food intake, using the most relevant search terms for the USA, UK, Canada, India and Australia. Daily and seasonal ISB rhythmicity were analysed using CircWave v. 1.4. Daily ISB data revealed a robust and significant sine waveform for general terms (e.g. ‘pizza delivery') and country-specific search terms (e.g. ‘just eat'). The pattern revealed clear evening double-peaks, occurring every day at 19.00 and 02.00. The patterns were consistent across search terms, days of the week and geographical locations, suggesting a common ISB rhythm that is not necessarily culture-dependent. Then, we conducted Cosinor v. 2.4 analyses to examine the daily amplitudes in ISB. The results indicated a non-significant linear increased from Monday to Sunday. Seasonal data did not show consistent significant ISB patterns. It is likely that two different human populations are responsible for the daily ‘early' and ‘late' evening ISB peaks. We propose that the major factor that contributes to the bimodal evening peak is age-dependent (e.g. adolescent, early adulthood versus midlife and mature adulthood) and a minor role for human chronotypes (e.g. late versus early). Overall, we present novel human appetitive behaviour for information seeking of food resources and propose that Internet-based search patterns reflect a biological rhythm of motivation for energy balance.

## 1. Introduction

The fundamental principle of adaptation is that the behaviours we observe have evolved, in part, from natural selection [1]. Successful foraging behaviour has been favoured by natural selection, which shaped innate, species-specific decision rules that maximize energy gain [2]. Across the animal kingdom, predator–prey interactions have resulted in several decisions that attempt to optimize the energetic gain per unit of time. The central place foraging theory aims to model hypotheses that concern animals collecting food and returning to a relatively fixed location [3]. Quantitative tests of foraging models generally include factors such as travel time, resource location and rates of prey capture/food intake. From an ethological perspective, foraging behaviours can be divided into two constructs: appetitive and consummatory behaviours. Appetitive behaviours include searching behaviours that are flexible and adaptive to the environment, whereas consummatory behaviours are innate and stereotyped [4]. Relatively less attention is given towards the species-dependent appetitive behaviours that precede food intake and energy gain and the time scales over which the behaviours are produced. Recent technological advancements, specifically Internet-based datasets, provide a unique and unparalleled capability to examine large-scale food-seeking appetitive behaviour. Here, we propose that information seeking behaviour (ISB) for food-associated search terms via Internet is a novel, human-specific appetitive behaviour that reflects food-related motivation.

Internet-based repositories have provided a significant resource to assess biological rhythms in human ISB [5]. ISB contained within Google Trends has been successfully applied to identify robust biological rhythms in Internet-based searches for many infections such as the flu [6–8] and common childhood diseases [9]. Importantly, the level of Google searches reflects medical reports derived from patient–physician interactions or cases of hospitalization (e.g. chicken pox [9–11]), indicating that Google Trends data have high biomedical validity. Thus, these ‘big-data' analyses permit the dissection of robust biological rhythms and provide a novel method to examine human information seeking associated with motivational aspects of foraging behaviour.

There is growing evidence to indicate clear variation in human circadian rhythmicity, commonly referred as ‘early' and ‘late' chronotypes [12]. The individual differences in chronotypes are maintained by both heritable [13] and socio-cultural factors [14]. Moreover, there are marked changes in chronotypes across the lifespan with the onset of puberty inducing a shift to late chronotypes [15] and subsequent return to early chronotypes in adulthood [16]. Large-scale analyses of dietary intake and preferences in female Japanese students revealed that late chronotypes tended to begin meals later, eat for a longer time and obtain higher percentage of energy from alcohol and fat [17]. Similarly, the daily food logs from adolescent individuals indicated that late chronotypes tended to drink more caffeinated drinks and fast food compared with early chronotypes [18].

The aim of this study was to characterize daily and seasonal rhythms in appetitive ISB for search terms associated with food and nutrition. Our objective was to interrogate Internet-based searches (i.e. Google Trends) over (i) two 1-week periods to assess daily rhythmicity and (ii) a 5-year period to examine seasonal variation. Owing to the limitations in number of countries in which reliable data could be collected, our analyses were restricted to only five countries. Our hypothesis was ISB for food-related terms would exhibit robust daily temporal variation and match stereotypical feeding patterns (i.e. morning, midday and evening meals).

## 2. Material and methods

### 2.1. Data collection

There has been a massive increase in the availability of Internet-based searches for food deliveries over the past decade (electronic supplementary material, figure S1). In order to assess daily and seasonal patterns of ISB, we collected data from Google Trends (www.google.co.uk/trends/) using methods described previously [6,9]. Seasonal data were surveyed over a 5-year period that spanned September 2011 to September 2016 and binned into monthly samples. For daily analyses, we sampled weekly data from two points in the annual cycle. These included 19–26 September 2016 and 14–20 March 2017. These two weeks provide the capability to reduce the potential for photoperiod effects to confound daily rhythms and to permit the comparison of Northern and Southern Hemispheres. The food-associated search terms interrogated included general terms (pizza delivery, Chinese delivery) and region specific terms (Just Eat, Panda Express, Swiggy, Zomato and Food Panda) (electronic supplementary material, figure S2). These terms were selected based on the Google Trends algorithm for ‘related queries' which identifies the most popular search queries for a selected geographical area (i.e. country). Search terms that did not exhibit an observable pattern due to low search rates were not analysed. The general terms were examined for the UK, the USA, Australia, Canada and India. Country-specific terms were Food Panda (USA, Canada), Just Eat (UK, Australia), Swiggy (India) and Zomato (India). To account for multiple time zones in the USA and Canada, Google Trends data were measured in each time zone independently and then averaged to form a national value. In brief, the information seeking value was determined by Google and is a normalized value based on the total number of searches conducted using the Google database over time. The values (0–100) are a per cent that is determined by dividing each monthly value by the highest month and multiplied by 100. The data are freely available online and did not require ethical approval.

### 2.2. Daily and seasonal information seeking behaviour waveform analyses

Google Trends data were analysed for daily and seasonal rhythms using CircWave v. 1.4. This program has been used previously to successfully analyse daily [19–21] and seasonal waveform [22]. The 1st harmonic analyses were conducted to assess statistical significance of a daily sine waveforms. Based on pilot data, we then conducted 4th harmonic analyses to evaluate whether the daily waveform conformed to four peaks (e.g. morning, noon and two evening searches). The centre of gravity (COG) was defined as the mean daily or seasonal time of all recorded information seeking searches. ANOVA analyses were conducted and all *F*- and *p*-values and centre of gravity were derived from CircWave v. 1.4. Statistical significance was determined at *p* < 0.001.

### 2.3. Determination of circadian amplitude

In order to assess whether ISB waveforms observed were consistent throughout every day of the week, we conducted linear regression analyses using the daily amplitudes. If the amount of Google information seeking increased dramatically on certain days, such as the weekend, then the amplitude should exhibit a significant increase on Saturday and Sunday (when oriented as Monday–Sunday). Alternatively, the absence of a significant regression would indicate that information seeking remained constant. Cosinor-based analyses are readily employed for the analyses of time series in chronobiology [23]. Amplitude of circadian waveforms was determined using Cosinor software developed by Roberto Refinetti (http://www.circadian.org/softwar.html). A linear regression was then conducted using SigmaPlot v. 13.0 to assess whether the level of food-related ISB terms changed over the week. Significance was determined at *p* < 0.001.

## 3. Results

### 3.1. Daily waveforms for general and specific terms

**Figure 1.**
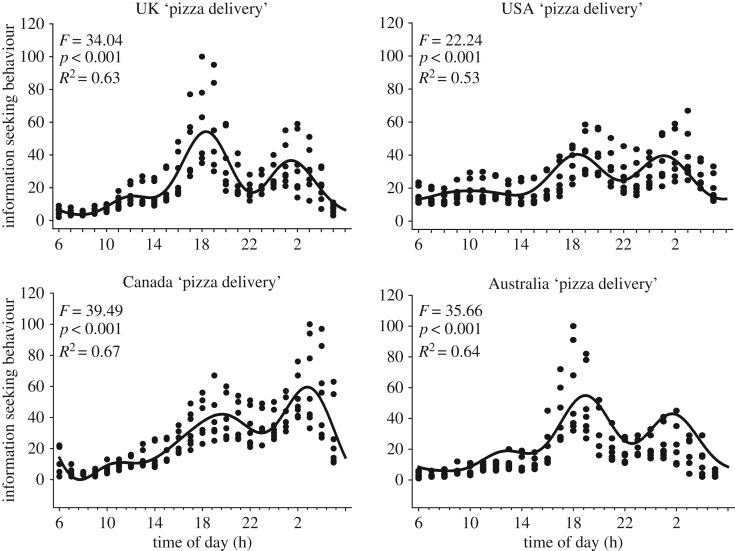
Daily ISB for general food-related search terms. There were robust daily oscillations for Internet searches for ‘pizza delivery' in the UK, USA, Canada and Australia. The daily waveform indicates best-fit patterns calculated using Circwave v. 1.4 analyses. Across all countries, there were large and consistent double evening peaks around 19.00 and 02.00.

**Figure 2.**
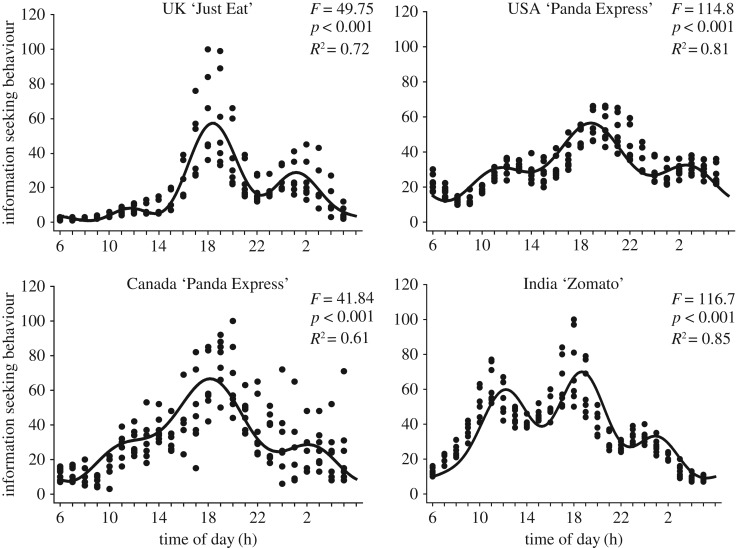
Daily ISB for country-specific food-related search terms. Robust daily waveforms were observed for ISB of keyword searches that targeted specific food resources such as ‘Just Eat', ‘Panda Express' and ‘Zomato'. The daily waveform indicates best-fit patterns calculated using Circwave 1.4 analyses. Similar to general search terms, there were clear double evening peaks around 19.00 and 02.00.

Circawave analyses revealed robust daily variation in ISB for both general and specific search terms. There were significant 1st harmonic waveforms for both ‘pizza delivery' and ‘Chinese delivery’ for Australia, Canada, the UK, India and the USA (*p* < 0.001; electronic supplementary material, table S1; figure 1). For Westernized countries, the COG ranged from 18.24 to 22.08 and 16.12 to 16.66 for India. The variability of the response data were no less than one-third (i.e. USA, ‘pizza delivery' *R*^2^ = 0.343) and no more than approximately two-thirds (i.e. Canada, ‘Chinese delivery’ *R*^2^ = 0.561). A similar pattern was observed for country-specific search terms. There were significant 1st harmonic waveforms for all countries (*p* < 0.001; electronic supplementary material, table S1; figure 2). The lack of a significant waveform for ‘Panda Express' in Australia probably reflects the lower use of the search term compared with ‘Just Eat'. The range for COG in Westernized countries were 17.42–2.02 for ‘Just Eat' in Canada and the USA and 16.12–17.40 in India (‘Swiggy’ and ‘Food Panda’). Overall, the variability in *R*^2^ broadened for specific terms, from 0.03 for Australia ‘Panda Express' to 0.64 for Canada ‘Just Eat'. These data demonstrate that ISB for food-related terms exhibits robust daily sine wave patterns for both general and specific search terms.

Next, we analysed the data using the 4th harmonic to determine whether the evening double peak could indicate potential early and late ISB patterns. Again, there were significant 4th harmonic waveforms for all search terms across the different countries (*p* < 0.001; figures 1 and 2; electronic supplementary material, table S1) with the exception of ‘Panda Express' in Australia (*p* = 0.39). Outside Australia, the *R*^2^ was larger compared with the 1st harmonic and ranged from 0.52 to 0.85 for general terms and 0.13–0.83 for specific terms. These data demonstrate that daily waveforms in ISB conform to the social norm for morning, noon and then an evening split with an early (i.e. 17.00–19.00) and late (i.e. 01.00—03.00) evening search. The data indicate that daily peak levels of ISB occur from 16.00 to 04.00, which contains a dual spike at approximately 19.00 and 02.00. To examine if ISB did not vary across time zones, we conducted a two-way ANOVA for factors (i) United States time zone (Eastern, Central, Mountain and Pacific) and (ii) daily peaks (07.00, 12.00, 19.00 and 02.00). Daily ISB did not vary across time zones in the USA (*p* > 0.13; electronic supplementary material, figure S3). There was a significant interaction (*p* < 0.05) and after Bonferroni's correction for multiple pairwise comparisons the only significant differences were (i) greater ISB during the morning phase in the Eastern compared with Mountain time zones and (ii) greater ISB in the Mountain compared with Eastern time zones during the late evening (electronic supplementary material, table S2). In order to determine the presence of country-specific ISB daily patterns, we conducted two-way ANOVA to compare peak phase (07.00, 12.00, 19.00 and 02.00) and country ISB. As expected, there was a significant time of day main effect (*p* < 0.001) and a main effect of country (*p* < 0.001; electronic supplementary material, figure S4). These data confirm the robust daily patterns revealed by the Circwave analyses and probably reflect the variation in Google usage across the respective countries. There was significant interaction (*p* < 0.001) and after Bonferroni's correction (*p* < 0.0025) there were relatively few pairwise differences (electronic supplementary material, table S3). Despite some country-specific time of day patterns, there were no clear cultural differences (e.g. India-specific phase peaks).

### 3.2. Weekly trend in daily information seeking behaviour for pizza delivery

**Figure 3.**
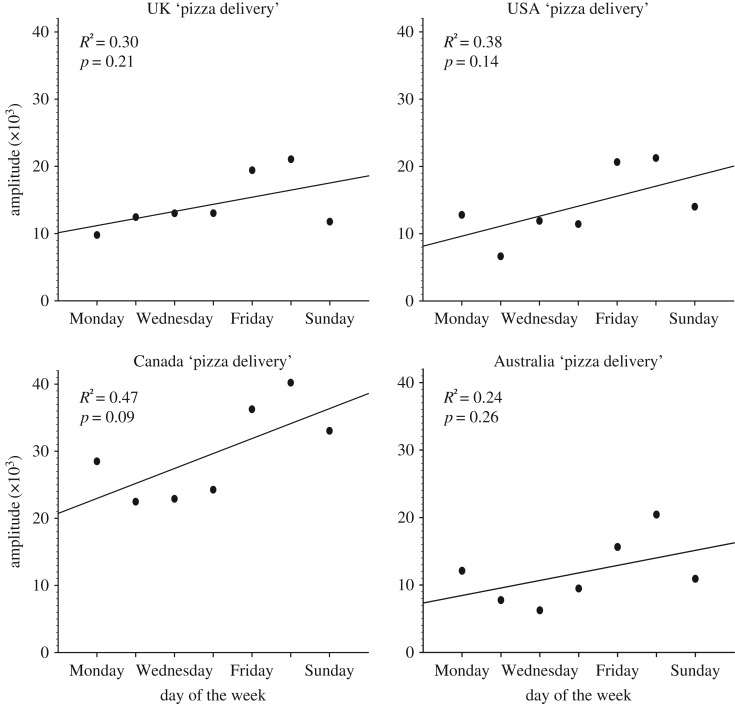
Linear regression of daily amplitude change of ISB for ‘pizza delivery'. Each point represents the daily amplitude for each day of the week, calculated using Cosinor 2.1. Overall, there was no significant increase for greater ISB from weekday to the weekend.

In order to determine if there was a linear increase in ISB from Monday to Sunday, we conducted a regression analyses using ‘pizza delivery'. Despite a positive linear trend for greater ISB later in the week (i.e. weekend); there were no significant patterns (*p* > 0.08; figure 3). The coefficient of determination across the countries was low and ranged from 0.24 (Australia) to 0.47 (Canada). These findings suggest that daily ISB is neither restricted nor greater during the weekend periods, but has a relatively consistent rate every day of the week.

### 3.3. Seasonal waveforms for general and specific terms

The overall pattern for seasonal ISB did not reveal a significant sine wave pattern (*p* > 0.01; electronic supplementary material, figure S5, table S4). However, for one general search term ‘Chinese delivery', there were robust seasonal patterns in Canada and the UK (*p* < 0.001; electronic supplementary material, table S4). Interestingly, the peak of ISB for ‘Chinese delivery' occurred in the middle of November (COG 22.62–23.21) for both Canada and the UK, respectively. The *R*^2^ for ‘Chinese delivery' was relatively low in Canada (0.36) and the UK (0.28). For all other search terms, the *R*^2^ value was less than 0.07. These data indicate that ISB for both general and specific food-related terms does not consistently show a seasonal waveform except for a couple perplexing instances.

## 4. Discussion

We examined how the variation in Google searches for food-related terms displays daily oscillations and identified large and consistent bi-modal evening peaks. ISB was observed in all countries studied and given the similar pattern between India and the other countries, we propose the patterns are not culturally dependent and instead are biologically motivated. This proposition is supported by the consistent daily rhythm and bi-modal evening peak each day of the week. Perhaps not surprisingly, ISB did not show consistent seasonal oscillations in food-related search terms and indicates that the motivation for Internet-based food resources is relatively constant across the year. To the best of our knowledge, these data are the first to describe Internet-based food searches, a modern appetitive foraging behaviour.

Overall, the results presented herein reveal novel human appetitive behaviour for food motivation on an international scale and the growing drive for Internet-based food resources (electronic supplementary material, figure S1) shows predictable daily rhythms (figures 1 and 2).

Google Trends database has been used before to identify biological patterns, notably seasonal rhythms of influenza [6–8] as well as other common childhood diseases [9]. Crucially, the patterns of ISB for influenza and chicken pox, in particular, were confirmed to forecast clinical observations [6,9]. The primary limitations of the current results are the inability to establish what diets were selected or whether ISB translated into food purchase and subsequent consumption. It was our goal to develop a central foraging model for Internet-based searching for food resources that incorporated ISB. Unfortunately, at this time, we are unable to capture several other critical factors such as resource composition (i.e. diet) and individual rates of food consumption [2,3]. At the current stage, the data indicate the presence of massive daily variation in appetitive ISB for general and specific food-related search terms that is probably driven by an underlying biological driver.

The daily bi-modal ISB peak was striking and suggests that either the same population searched for two evening meals or these peaks represent distinct populations. Although we are unable to confirm the characteristics of individuals that perform ISB for food-related terms, we propose that it is unlikely that the same population conducts ISB twice (i.e. early and late evening peaks) every day of the week. In order to determine whether the same individuals searched for food during the two evening meals would require access to Internet Protocol (IP) address information that is not provided by Google Trends. We propose that the level of individual appetitive behaviour for multiple searches captured in Google Trends is probably minor and instead, support the conjecture that the majority of the two evening searches reflect different individuals.

One tantalizing hypothesis that would account for the distinct evening peaks is centred on early and late human chronotypes [12]. This conjecture is probably too simplistic, as early and late chronotypes are a relatively minor proportion of the population and the majority of ISB is driven by moderate chronotypes [12]. Moreover, night eating syndrome could also contribute to the late evening ISB peak, although it is probably relatively minor due to the low 1.5–3% of the population [17,24–26]. Alternatively, the bimodal peak could reflect age or lifestyle populations, such as post-secondary students (e.g. college/university) or an age-dependent plasticity in chronotypes [15,16]. To examine whether the double spike could be determined by student age-populations, we assessed two different cities. Champaign–Urbana, Illinois, where the student population encompasses 21.8% of the population was compared to Chicago, Illinois, where the student population makes up approximately 11% of the population [27]. The observed pattern indicated that the phenomenon is not purely explained by large populations of students, as similar ISB rhythms were present in both demographics (electronic supplementary material, figure S6). This rather restricted view does not take into consideration adolescent-aged, non-higher education and early professional age ranges. Unfortunately, we were unable to identify a geographical region that could separate the various age-demographics and had sufficient Google data to further investigate age-dependent search patterns.

The appearance of daily rhythms across diverse cultures and countries supports the probability for an underlying biological driver for ISB rhythmicity. This is emphasized by the consistent ISB daily rhythms that exhibit a double evening spike in India (figure 2; electronic supplementary material, tables S1 and S2). There are slight differences between India and the other countries, such as a higher level of midday ISB and lower ‘evening' ISB. Therefore, daily rhythms in food-related ISB cannot solely be accounted for by Western cultures. ISB patterns that would support a culturally driven behaviour would include phase peaks during major annual festivals (e.g. Thanksgiving, Christmas and Diwali). The absence of ISB during cultural events distinct from other times of the year, or between countries supports the notion for a biological basis of this appetitive behaviour. Indeed, a large number of diverse cultures would aid the conjecture that appetitive ISB is a common feature of human behaviour. Based on the massive dataset contained within Google Trends, we speculate that the expansion of Internet access into other countries and cultures will continue to reflect the ISB rhythms in the present paper.

In summary, our results show that appetitive ISB for food-related terms exhibit robust daily oscillations. The daily waveform was consistent across days of the week, cultures and did not vary with seasons. We proposed that an underlying biological mechanism drives ISB patterns in human appetitive behaviours as analysed from a massive big-data source, Google Trends. Appetitive ISB for food-related search terms is probably an output of multiple interconnected neural structures (e.g. food-entrainable oscillator [28]) that includes circadian properties and is entrained by hormone signalling. The methods described herein provide a novel means to examine biological rhythms in human appetitive behaviours and the opportunity to expand the capability to examine a wide range of human motivations.

## Supplementary Material

Figure S1 2010-2017 increased use of Google ISB

## Supplementary Material

Figure S2 Google terms used to examine food related ISB

## Supplementary Material

Figure S3 Daily ISB in four time zones in the United States

## Supplementary Material

Figure S4 Daily ISB for country specific Google search terms

## Supplementary Material

Figure S5 Seasonal ISB waveform across countries

## Supplementary Material

Figure S6

## Supplementary Material

Table S1: Statistical summary of daily analyses for general and specific terms

## Supplementary Material

Table S2 - Significant interaction US time zones by time of day pairwise comparisons

## Supplementary Material

Table S3 - Significant interactions of country specific terms and time of day pairwise comparisons.

## Supplementary Material

Table S4: Statistical summary of seasonal analyses for general and specific terms

## Data accessibility

The data are freely available online in Google Trends (www.google.co.uk/trends/).
