# Supplementary material for: Appetitive information seeking behaviour reveals robust daily rhythmicity for Internet-based food-related keyword searches
Source: R Soc Open Sci. 2018 Jul 25;5(7):172080. doi: 10.1098/rsos.172080 (PMC6083665; doi:10.1098/rsos.172080)
Supplement: Figure S1 2010-2017 increased use of Google ISB [file rsos172080supp1.pdf]

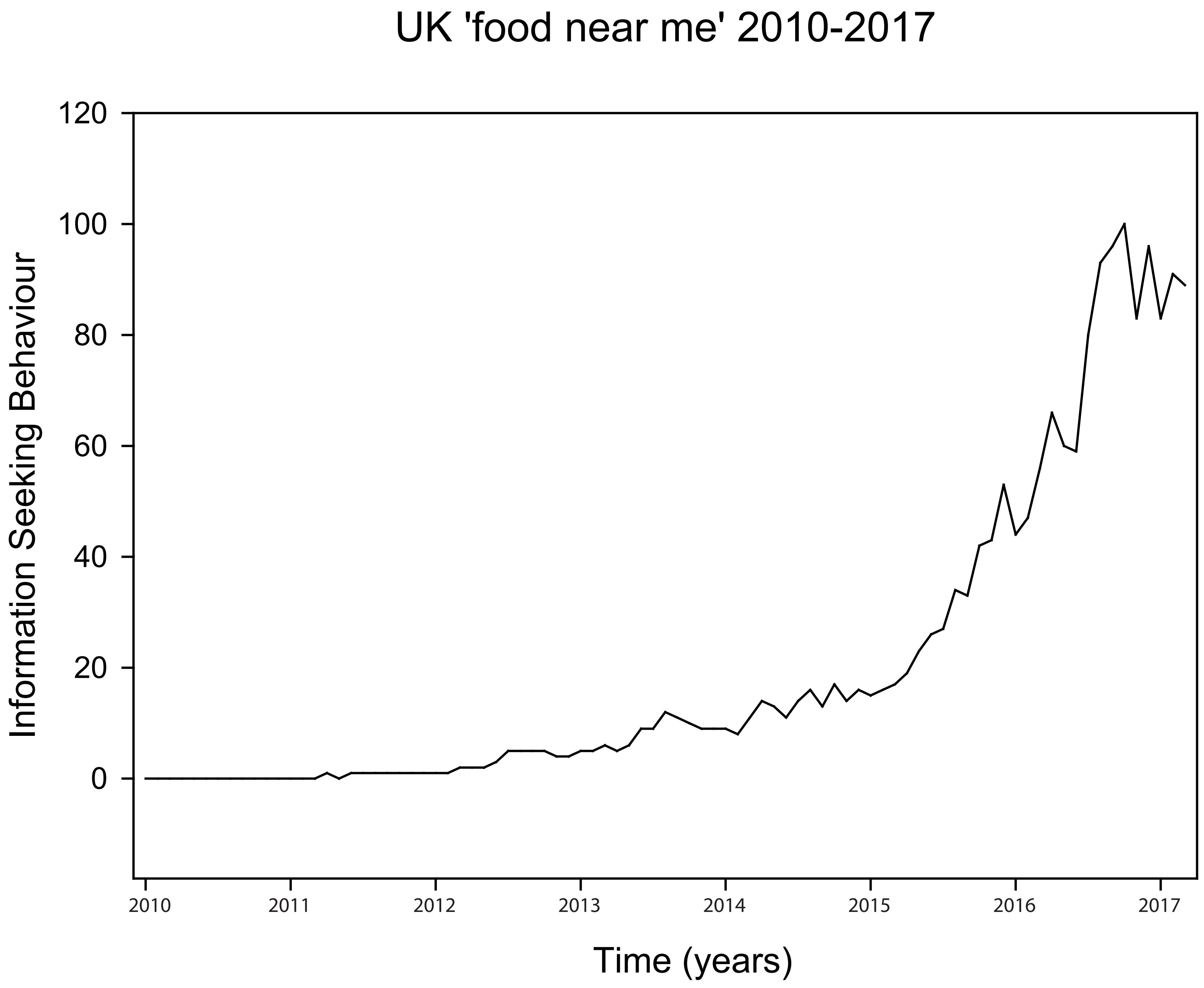

Figure S1: Increase in global searches of “delivery near me” over 2004-2017. Line of best fit denotes an exponential increase.
