# Supplementary material for: Appetitive information seeking behaviour reveals robust daily rhythmicity for Internet-based food-related keyword searches
Source: R Soc Open Sci. 2018 Jul 25;5(7):172080. doi: 10.1098/rsos.172080 (PMC6083665; doi:10.1098/rsos.172080)
Supplement: Figure S2 Google terms used to examine food related ISB [file rsos172080supp2.pdf]

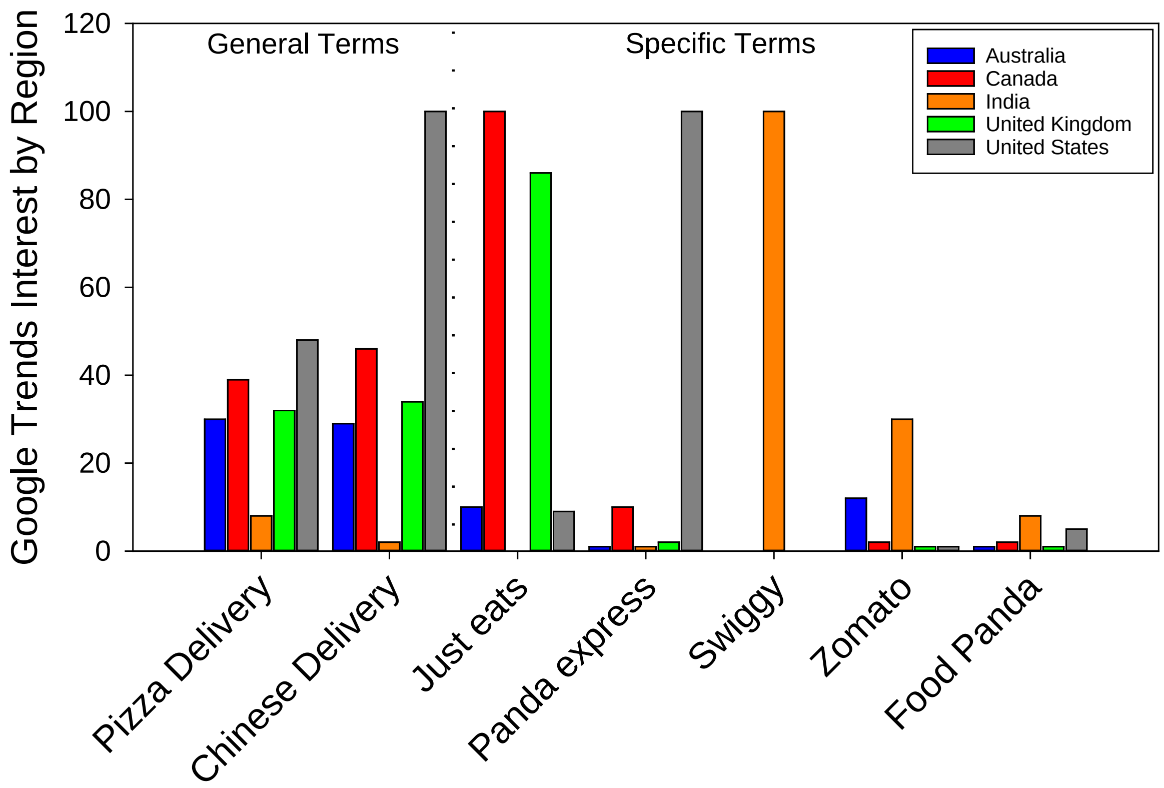

Figure S2 – The relative interest for Google search key words across countries that were classified as 'General' (i.e. Pizza Delivery & Chinese Delivery) or 'Specific' (i.e. Just eats, Panda express, Swiggy, Zomato & Food panda). General terms were classified as those that were observed across most countries and had a relatively equal distribution of interest as determined by Google database algorithms. India appears to use General terms at a very low level compared to the other countries that were assessed. The Specific terms were selected from the list of Google defined 'Related search terms' produced after analyses of trends in General terms. Each Specific term showed a country-dependent pattern, for example, Zwiggy was only used for Google searches in India.
