# Supplementary material for: Appetitive information seeking behaviour reveals robust daily rhythmicity for Internet-based food-related keyword searches
Source: R Soc Open Sci. 2018 Jul 25;5(7):172080. doi: 10.1098/rsos.172080 (PMC6083665; doi:10.1098/rsos.172080)
Supplement: Figure S3 Daily ISB in four time zones in the United States [file rsos172080supp3.pdf]

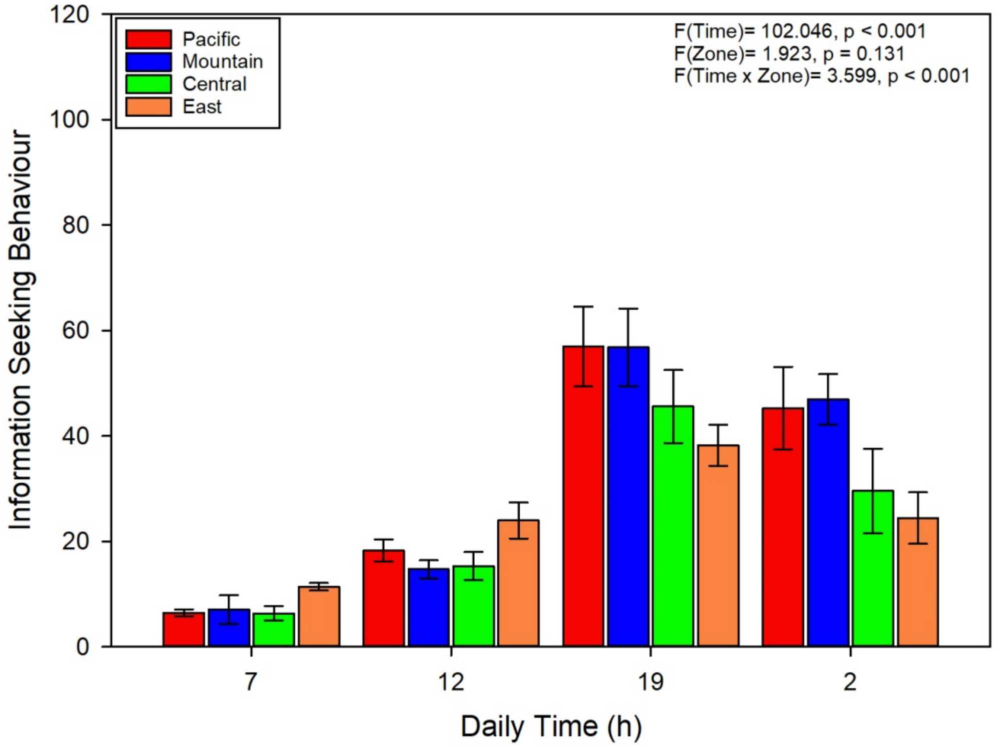

Figure S3 – Daily variation in phase peaks across the four time zones in the United States. There was a significant main effect for time-of-day in Google searches for 'just eats'. There was no significant difference between time zones suggesting that the frequency of Google searches was no dependent on geographical location within the United States. There was a significant interaction (see Table S2). The Eastern time zone had greater information seeking behaviour during the morning phase compared to Mountain time zone. The Mountain time zone had significantly higher ISB during the late evening compared to the Eastern time zone. Data are mean and standard error.
