# Supplementary material for: Appetitive information seeking behaviour reveals robust daily rhythmicity for Internet-based food-related keyword searches
Source: R Soc Open Sci. 2018 Jul 25;5(7):172080. doi: 10.1098/rsos.172080 (PMC6083665; doi:10.1098/rsos.172080)
Supplement: Figure S4 Daily ISB for country specific Google search terms [file rsos172080supp4.pdf]

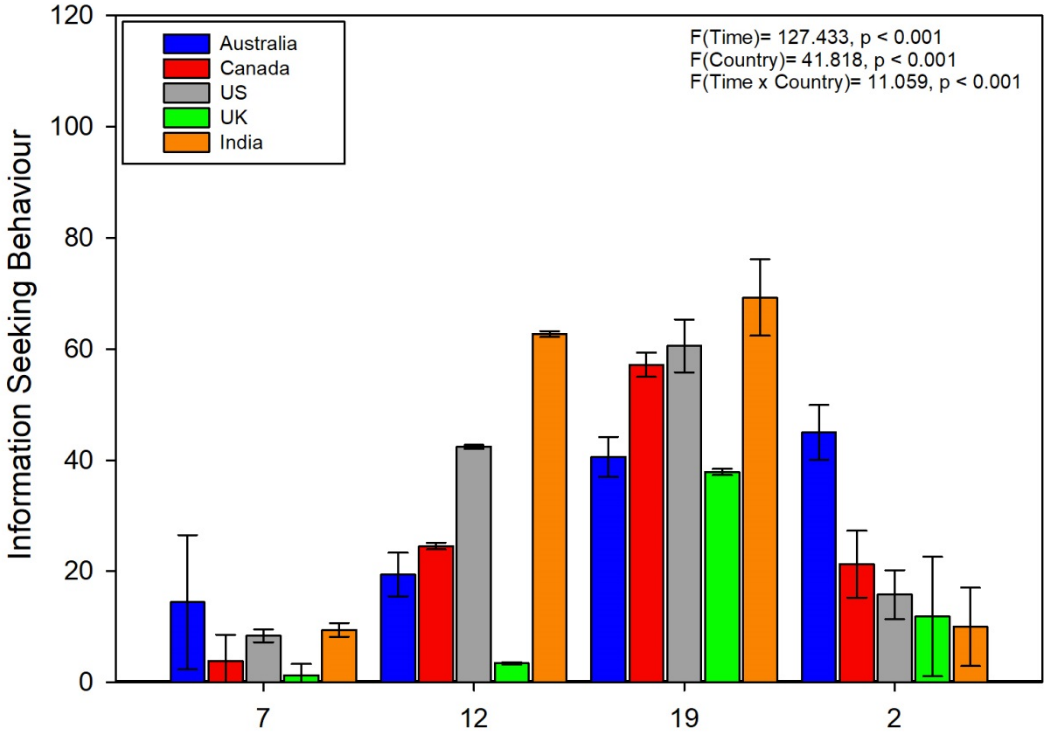

Figure S4 – Daily variation in country specific information seeking behaviour (ISB). There was a significant difference in the time of day for ISB with higher usage during the early evening phase (19h). There was significant main effect of country, which is likely driven by the population size with access to internet capable devices. There was a significant interaction for country specific by time of day ISB pattern (See Table S3). The United States and India had generally higher ISB during the noon and early evening times of day. Australia displayed significantly higher rates of ISB during the late evening. Data are mean and standard error.
