# Supplementary material for: Appetitive information seeking behaviour reveals robust daily rhythmicity for Internet-based food-related keyword searches
Source: R Soc Open Sci. 2018 Jul 25;5(7):172080. doi: 10.1098/rsos.172080 (PMC6083665; doi:10.1098/rsos.172080)
Supplement: Figure S5 Seasonal ISB waveform across countries [file rsos172080supp5.pdf]

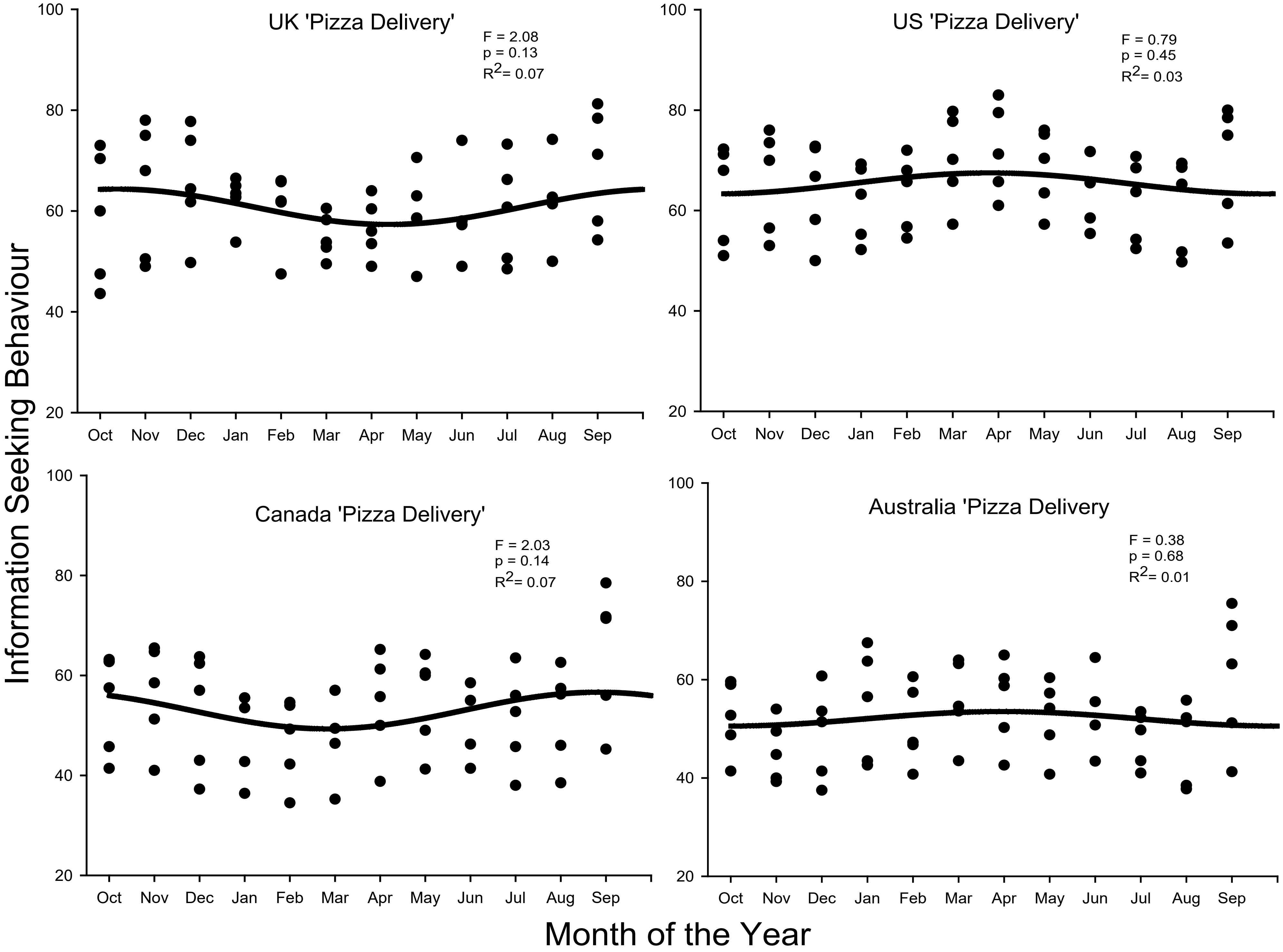

Figure S2: Seasonal information seeking behaviour for "pizza delivery" over 2011-2016 for the Australia, Canada, the US and the UK. Circwave 1.4 analysis waveform indicates no seasonal pattern.
