# Supplementary material for: Appetitive information seeking behaviour reveals robust daily rhythmicity for Internet-based food-related keyword searches
Source: R Soc Open Sci. 2018 Jul 25;5(7):172080. doi: 10.1098/rsos.172080 (PMC6083665; doi:10.1098/rsos.172080)
Supplement: Figure S6 [file rsos172080supp6.pdf]

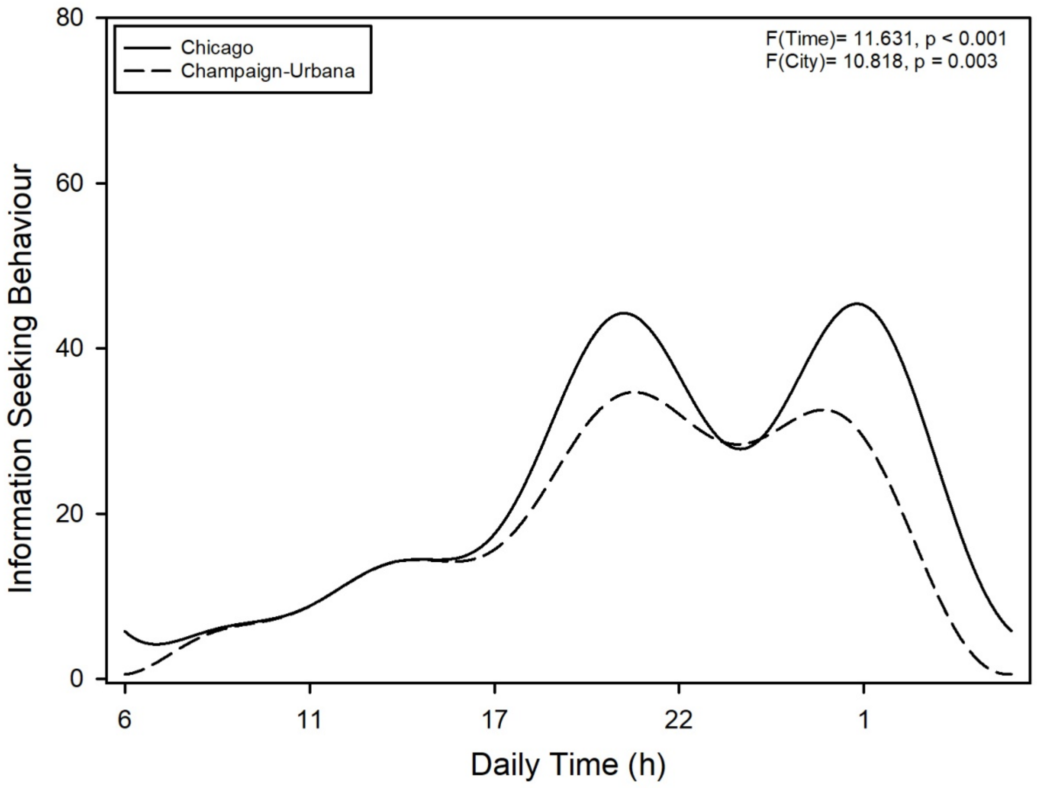

Figure S6 – Daily variation of ISB for ‘just eats’ in Chicago and Champaign-Urbana. There was a significant daily change in Google searches with phase peaks during the morning (7h), noon (12h), and early and late evening (19h and 2h, respectively). There was a significant main effect of city. The higher frequency of ISB in Chicago compared to Champaign-Urbana populations is driven likely due to the substantial differences in city populations. Overall, the daily waveforms between the two cities suggest a similar biological basis that drives internet-based searches. Data were derived from Circawave analyses.
