# Supplementary material for: Appetitive information seeking behaviour reveals robust daily rhythmicity for Internet-based food-related keyword searches
Source: R Soc Open Sci. 2018 Jul 25;5(7):172080. doi: 10.1098/rsos.172080 (PMC6083665; doi:10.1098/rsos.172080)
Supplement: Table S1: Statistical summary of daily analyses for general and specific terms [file rsos172080supp7.pdf]

Table S1: Statistical summary of daily analyses for general and specific terms

Country	Term	Harmonic	COG	Mean	SD	df	F-stat	p-value	R^2
Australia	Pizza Delivery	1	19.65	17.83	17.42	2	59.21	< 0.001	0.42
		4				8	35.66	< 0.001	0.64
	Chinese Delivery	1	18.24	12.32	17.53	2	51.18	< 0.001	0.38
		4				8	54.71	< 0.001	0.73
	Just Eat	1	22.74	31.59	18.36	2	8.28	< 0.001	0.09
		4				8	4.20	< 0.001	0.17
	Panda Express	1	0.09	15.69	14.85	2	2.70	0.07	0.03
		4				8	1.07	0.39	0.05
Canada	Pizza Delivery	1	22.08	27.16	20.83	2	65.62	< 0.001	0.44
		4				8	39.49	< 0.001	0.67
	Chinese Delivery	1	18.92	21.41	21.34	2	105.51	< 0.001	0.56
		4				8	36.82	< 0.001	0.65
	Just Eat	1	17.42	27.25	21.80	2	148.24	< 0.001	0.64
		4				8	74.16	< 0.001	0.79
	Panda Express	1	17.97	32.80	21.48	2	86.34	< 0.001	0.51
		4				8	32.94	< 0.001	0.62
United Kingdom	Pizza Delivery	1	20.10	22.54	18.20	2	54.22	< 0.001	0.40
		4				8	34.04	< 0.001	0.63
	Chinese Delivery	1	19.17	13.71	15.76	2	53.46	< 0.001	0.39
		4				8	33.26	< 0.001	0.63
	Just Eat	1	20.01	18.62	19.29	2	73.78	< 0.001	0.47
		4				8	49.75	< 0.001	0.72
	Panda Express	1	23.17	21.28	16.56	2	19.06	< 0.001	0.19
		4				8	8.58	< 0.001	0.30
United States	Pizza Delivery	1	21.40	24.92	12.31	2	43.07	< 0.001	0.34
		4				8	22.24	< 0.001	0.53
	Chinese Delivery	1	19.30	19.79	12.14	2	93.7	< 0.001	0.53
		4				8	51.75	< 0.001	0.72
	Just Eat	1	2.027	19.29	5.193	2	12.76	< 0.001	0.13
		4				8	6.79	< 0.001	0.25
	Panda Express	1	18.87	31.75	13.02	2	124.8	< 0.001	0.60
		4				8	86.7	< 0.001	0.81
India	Zomato	1	16.66	36.74	19.49	2	142.24	< 0.001	0.63
		4				8	116.7	< 0.001	0.85
	Swiggly	1	16.12	27.22	20.98	2	100.36	< 0.001	0.55
		4				8	99.65	< 0.001	0.83
	FoodPanda	1	17.40	25.60	20.31	2	54.21	< 0.001	0.40
		4				8	57.3	< 0.001	0.74
