# Supplementary material for: Appetitive information seeking behaviour reveals robust daily rhythmicity for Internet-based food-related keyword searches
Source: R Soc Open Sci. 2018 Jul 25;5(7):172080. doi: 10.1098/rsos.172080 (PMC6083665; doi:10.1098/rsos.172080)
Supplement: Table S2 - Significant interaction US time zones by time of day pairwise comparisons [file rsos172080supp8.pdf]

Table S2 – Significant interaction US time zones by time of day pairwise comparisons.

Comparisons for factor: **Zone within 2**

| **Comparison** | **Diff of Means** | **P** |
|---|---|---|
| Mountain vs. East | 0.326 | 0.002* |
| Mountain vs. Central | 0.263 | 0.013 |
| Mountain vs. Pacific | 0.0387 | 0.711 |
| Pacific vs. East | 0.287 | 0.007 |
| Pacific vs. Central | 0.225 | 0.033 |
| Central vs. East | 0.0622 | 0.551 |

Comparisons for factor: **Zone within 7**

| **Comparison** | **Diff of Means** | **P** |
|---|---|---|
| East vs. Mountain | 0.379 | <0.001* |
| East vs. Central | 0.303 | 0.004 |
| East vs. Pacific | 0.254 | 0.016 |
| Pacific vs. Mountain | 0.125 | 0.232 |
| Pacific vs. Central | 0.0491 | 0.638 |
| Central vs. Mountain | 0.0759 | 0.467 |

Comparisons for factor: **Zone within 12**

| **Comparison** | **Diff of Means** | **P** |
|---|---|---|
| East vs. Mountain | 0.200 | 0.057 |
| East vs. Central | 0.198 | 0.060 |
| East vs. Pacific | 0.102 | 0.328 |
| Pacific vs. Mountain | 0.0982 | 0.347 |
| Pacific vs. Central | 0.0955 | 0.360 |
| Central vs. Mountain | 0.00264 | 0.980 |

Comparisons for factor: **Zone within 19**

| **Comparison** | **Diff of Means** | **P** |
|---|---|---|
| Pacific vs. East | 0.165 | 0.115 |
| Pacific vs. Central | 0.103 | 0.324 |
| Pacific vs. Mountain | 0.00136 | 0.990 |
| Mountain vs. East | 0.164 | 0.118 |
| Mountain vs. Central | 0.102 | 0.330 |
| Central vs. East | 0.0622 | 0.551 |

* denotes significant pairwise after Bonferroni correction (P < 0.003)
