# Supplementary material for: Appetitive information seeking behaviour reveals robust daily rhythmicity for Internet-based food-related keyword searches
Source: R Soc Open Sci. 2018 Jul 25;5(7):172080. doi: 10.1098/rsos.172080 (PMC6083665; doi:10.1098/rsos.172080)
Supplement: Table S3 - Significant interactions of country specific terms and time of day pairwise comparisons. [file rsos172080supp9.pdf]

Table S3 – Significant interactions of country specific terms and time of day pairwise comparisons.

Comparisons for factor: **Country within 2**

**Comparison**	**Diff of Means**	**P**
Australia vs. India	0.587	<0.001*
Australia vs. UK	0.524	<0.001*
Australia vs. US	0.373	0.001*
Australia vs. Canada	0.310	0.007
Canada vs. India	0.277	0.015
Canada vs. UK	0.214	0.060
Canada vs. US	0.0636	0.573
US vs. India	0.213	0.061
US vs. UK	0.150	0.184
UK vs. India	0.0627	0.578

Comparisons for factor: **Country within 7**

**Comparison**	**Diff of Means**	**P**
Australia vs. UK	0.916	<0.001*
Australia vs. Canada	0.449	<0.001*
Australia vs. US	0.0803	0.477
Australia vs. India	0.0320	0.776
India vs. UK	0.884	<0.001*
India vs. Canada	0.417	<0.001*
India vs. US	0.0483	0.669
US vs. UK	0.836	<0.001*
US vs. Canada	0.368	0.001*
Canada vs. UK	0.467	<0.001*

Comparisons for factor: **Country within 12**

**Comparison**	**Diff of Means**	**P**
India vs. UK	1.282	<0.001*
India vs. Australia	0.580	<0.001*
India vs. Canada	0.404	<0.001*
India vs. US	0.159	0.161
US vs. UK	1.123	<0.001*
US vs. Australia	0.421	<0.001*
US vs. Canada	0.246	0.031
Canada vs. UK	0.877	<0.001*
Canada vs. Australia	0.175	0.122
Australia vs. UK	0.702	<0.001*

Comparisons for factor: **Country within 19**

**Comparison**	**Diff of Means**	**P**
India vs. UK	0.348	0.002*
India vs. Australia	0.240	0.035
India vs. Canada	0.0833	0.461
India vs. US	0.0517	0.647
US vs. UK	0.296	0.010
US vs. Australia	0.188	0.098
US vs. Canada	0.0316	0.779
Canada vs. UK	0.265	0.020
Canada vs. Australia	0.156	0.167
Australia vs. UK	0.108	0.337

* denotes significant pairwise after Bonferroni correction ($P < 0.0025$)
