# Supplementary material for: Appetitive information seeking behaviour reveals robust daily rhythmicity for Internet-based food-related keyword searches
Source: R Soc Open Sci. 2018 Jul 25;5(7):172080. doi: 10.1098/rsos.172080 (PMC6083665; doi:10.1098/rsos.172080)
Supplement: Table S4: Statistical summary of seasonal analyses for general and specific terms [file rsos172080supp10.pdf]

Table S4: Statistical summary of seasonal analyses for general and specific terms

Country	Term	Harmonic	COG	Mean	SD	df	F-stat	p-value	R2
Australia	Pizza Delivery	1	7.39	52.02	9.10	2	0.38	0.68	0.01
	Chinese Delivery		6.31	54.27	6.96	2	0.70	0.50	0.02
	Just Eat		7.48	46.18	9.94	2	0.57	0.56	0.02
	Panda Express		4.10	37.04	6.93	2	0.21	0.81	0.01
Canada	Pizza Delivery	1	23.44	52.96	10.12	2	2.03	0.14	0.07
	Chinese Delivery		22.62	47.75	8.54	2	15.99	< 0.001	0.36
	Just Eat		3.18	37.23	18.12	2	0.75	0.47	0.03
	Panda Express		5.11	42.98	16.54	2	0.11	0.89	0.00
United Kingdom	Pizza Delivery	1	1.87	60.87	9.56	2	2.08	0.13	0.07
	Chinese Delivery		23.21	56.91	5.28	2	10.97	< 0.001	0.28
	Just Eat		4.76	49.98	12.87	2	0.03	0.97	0.00
	Panda Express		5.28	37.39	9.36	2	1.48	0.23	0.05
United States	Pizza Delivery	1	7.65	65.39	9.03	2	0.79	0.45	0.03
	Chinese Delivery		0.04	64.93	6.29	2	4.26	0.02	0.13
	Just Eat		7.26	61.36	8.76	2	4.77	0.01	0.15
	Panda Express		6.46	58.25	12.09	2	0.64	0.53	0.02
India	Zomato	1	11.22	51.98	22.27	2	0.44	0.65	0.02
	Swiggy		11.11	5.35	9.43	2	0.63	0.54	0.02
	FoodPanda		11.75	15.92	20.02	2	0.56	0.57	0.02
